# Supplementary material for: Interferometric excitation fluorescence lifetime imaging microscopy
Source: Nat Commun. 2024 Sep 13;15:8019. doi: 10.1038/s41467-024-52333-2 (PMC11399241; doi:10.1038/s41467-024-52333-2)
Supplement: Supplementary file 1 — Supplementary Information [file 41467_2024_52333_MOESM1_ESM.pdf]

Supplementary Information to:

Interferometric Excitation Fluorescence Lifetime Imaging Microscopy

Pavel Malý[1*], Dita Strachotová[1], Aleš Holoubek[2], Petr Heřman[1]

[1]*Faculty of Mathematics and Physics, Institute of Physics, Charles University, Prague, Czech Republic*

[2]*Department of Proteomics, Institute of Hematology and Blood Transfusion, Prague, Czech Republic*

**pavel.maly@mff.cuni.cz*

S1. Optical setup

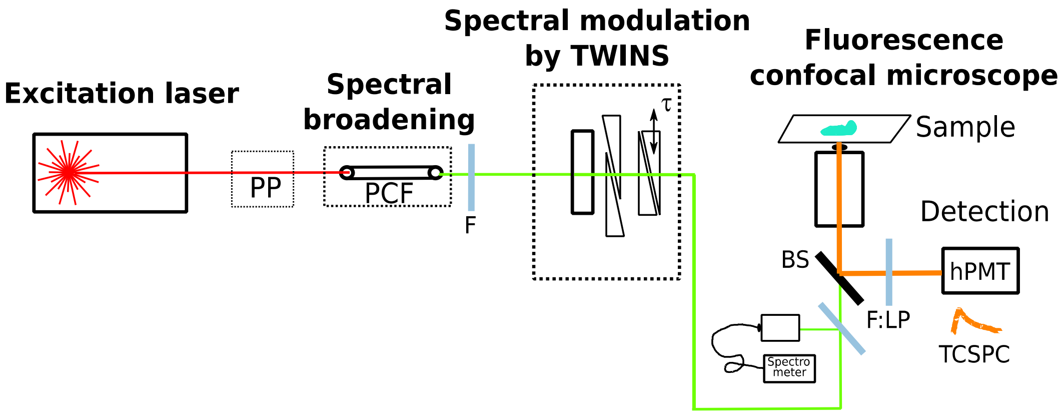

Fig. S1 Experimental setup. Excitation laser (Coherent Chameleon Ultra II) pulses (~150 fs, 760 nm, 4 MHz, 4 nJ after pulse picker (PP, PulseSelect from APE) and attenuation) seed the photonic crystal fibre (PCF, SUP-2-125, custom-adjusted in collaboration with Photonics Bretagne), which generates spectrally broad (~450 nm to 700 nm after long- and short-pass filtering (F, Thorlabs)) spectrum. The broadband pulses enter the home-built TWINS interferometer used for creating a double-pulse with a variable inter-pulse delay τ. Fraction of the pulses is split off and coupled by a 10x objective to a fibre spectrometer (SR-2, Ocean Insight), where the spectrum is used for reference and calibration. The pulses are further attenuated and coupled into a commercial confocal scanning fluorescence microscope (Olympus iX83 with FV1200 scanner), where they excite the sample by apochromatic objective (Olympus). The fluorescence emission is split off by a beam splitter, the residual excitation is removed by a long-pass filter (F:LP, Thorlabs), and detected by hybrid photomultiplier (PMA hybrid 40, PicoQuant) counted by a TCSPC module (TimeHarp 260 PICO, PicoQuant).

Our experimental setup, already briefly described in the Methods in the main text, is schematically shown and described in Fig. S1. We use a custom-built excitation coupled to a commercial microscope with FLIM. To facilitate high-enough photon counts per second that allow fast image scanning and sensitive acquisition of weak fluorescence signals at low excitation pulse energies, FLIM measurements are typically done at MHz pulse repetition rates. To create spectrally ultrabroadband pulses at such repetition frequencies we utilize a photonic crystal fiber (PCF), custom-made in collaboration with Photonics Bretagne, see details in section S2. Pumped by a Ti:Sapphire oscillator (Chameleon Ultra II, Coherent), our PCF spectrum ranges from 400 nm to 1000 nm, with a typical repetition rate of 4 MHz to 8 MHz. Note, that alternative fiber-based white light pulsed lasers with similar parameters are commercially available. The spectrum is restricted to the desired excitation window by a shortpass filter at the long-wavelength edge of the absorption. The pulse pair for spectral modulation of the excitation is produced interferometrically. We use a custom-built version of the birefringent common-path interferometer called TWINS[1,2], that features excellent both phase stability and beam pointing stability. Our TWINS uses AR-coated α-BBO wedges (FOCTek) of the same parameters as in Ref. [2], mounted on a piezo stage (V-408, PI) that allows rapid and precise scanning of the delay time τ. For the detailed principle and setup of the TWINS interferometer we refer the reader to Refs. [1,2]. Briefly,

the delay between the ordinary and extraordinary waves in the birefringent α-BBO crystal is used to separate the pulse into two time-delayed replicas. Translation of a pair of wedges into the beam alters the thickness of the material in which the pulses travel with different refractive index, and thus changes the inter-pulse delay τ. The pulse pairs are free-space coupled into the commercial scanning confocal fluorescence microscope (iX83 with FV1200 scanner, Olympus) where they are used to excite the sample, using an apochromatic objective (Olympus UPlanSApo 10x, 0.4NA for the Oxo VI, Olympus UPlanSApo 40x, 0.95NA, for the Cy3–Cy5-labeled beads and water immersion Olympus UplanSApo 60xW 1.2NA for the HEK-293T cells). The fluorescence is detected through a confocal pinhole in a de-scanned port by a cooled hybrid photomultiplier tube (PMA Hybrid 40, PicoQuant), counted by a TCSPC module (TimeHarp 260, PicoQuant). The acquisition was realized by SymphoTime software (PicoQuant), triggered by scanning software of the microscope (Olympus). The microscope scan is triggered by a custom-made program in Matlab (Mathworks), that coordinates the experiment and controls the other instruments as well (TWINS, fiber spectrometer). Reference excitation spectral profile on the input port of the microscope is measured during the acquisition by a fiber spectrometer (SR-2, Ocean Insight).

S2. Ultrabroadband interferometry

The broadband white light continuum is generated in a photonic crystal fiber (PCF)[3], custom-made in collaboration with Photonics Bretagne. The SUP-2-125 fiber is about 12 cm long, and, to prevent thermal damage to the fiber, sealed endcaps were added by Photonics Bretagne at both ends of the PCF, preventing contamination and, at the same time, protecting against the surface burn since the focus lies on the end-cap–core interface and is not exposed to air. The PCF is pumped by about 4 nJ pulses, centered at 760 nm, at repetition rate of 4 MHz, producing spectrum typically ranging from 450 nm to 1000 nm. The spectrum is restricted by long- and short-pass filters to the desired spectral range.

Here, we describe the calibration procedure for the excitation by our home-built TWINS[1] interferometer based on birefringent wedges with variable insertion into the beam. In the TWINS interferometer, the delay between the two pulses is scanned by a stage translating a pair of birefringent wedges[1,2]. For each step of the stage, the modulated spectrum is recorded just before entering the microscope, using a back-reflection from an ND filter and 10x objective coupling into fiber spectrometer. The resulting spectral interference is depicted in Fig. S2a.

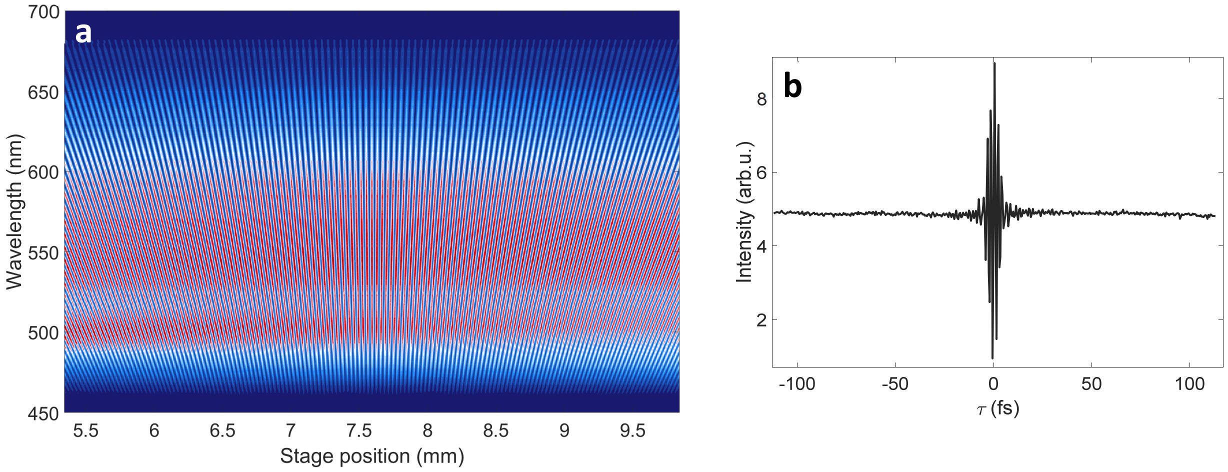

Figure S2 Spectral interferometry for TWINS calibration and reference spectrum. a) spectral interference of the pulse pair generated by the interferometer, as a function of the TWINS stage position. b) Spectrally integrated interferogram of the same scan, already with axis translated into time delay τ.

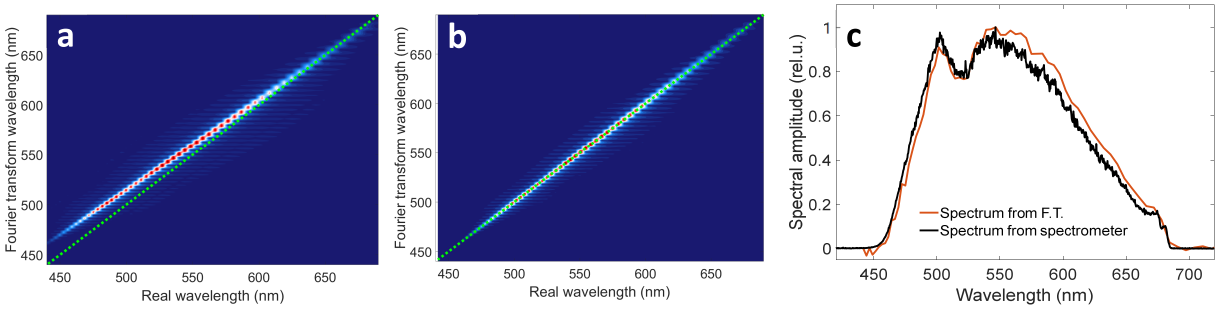

Figure S3 Calibration of the interferometer. a) uncalibrated Fourier-transformed spectra stack, b) the same but with the F.T. wavelength axis calibrated as described in the text. The dotted green line indicates the desired wavelength correspondence. c) Recovered spectrum from the interferogram in Fig. S2b (orange), compared to the spectrometer-acquired spectrum (black).

Clearly, the tilt of the fringes is symmetric around a specific position of the stage just above 7.5 mm, which corresponds to the $\tau = 0$ point. To determine this point more precisely, we integrate the spectrum over the detection wavelength, obtaining the interferogram (Fig. S2b). Fourier-transform of this interferogram has to yield the laser spectrum. We thus guess the $\tau = 0$ point from the maximum of the interferogram and iteratively adjust it, taking for each point the real part of the Fourier transform. The true $\tau = 0$ point will be the one for which is the spectrum without modulation resulting from delay phase twists and has maximum area under its curve. Having thus found the $\tau = 0$ point, we guess the stage step size as 50 fs/mm, getting the time delay axis used for the interferogram in Fig. S2b. The scan step size and length are given by the Nyquist limit, determined by the lowest wavelength in the spectrum and the desired spectral resolution. In our measurement, we scan the interferogram symmetrically around $\tau = 0$ with stage steps of 0.015 mm (0.75 fs), measuring 301 steps (from -112.5 fs to 112.5 fs).

To obtain the spectral calibration, we plot the spectral interference in Fourier domain correlating λ_{FT} and λ_{real} from the spectrometer (Fig. S3a). Like this, we get one wavelength point (around 650 nm)

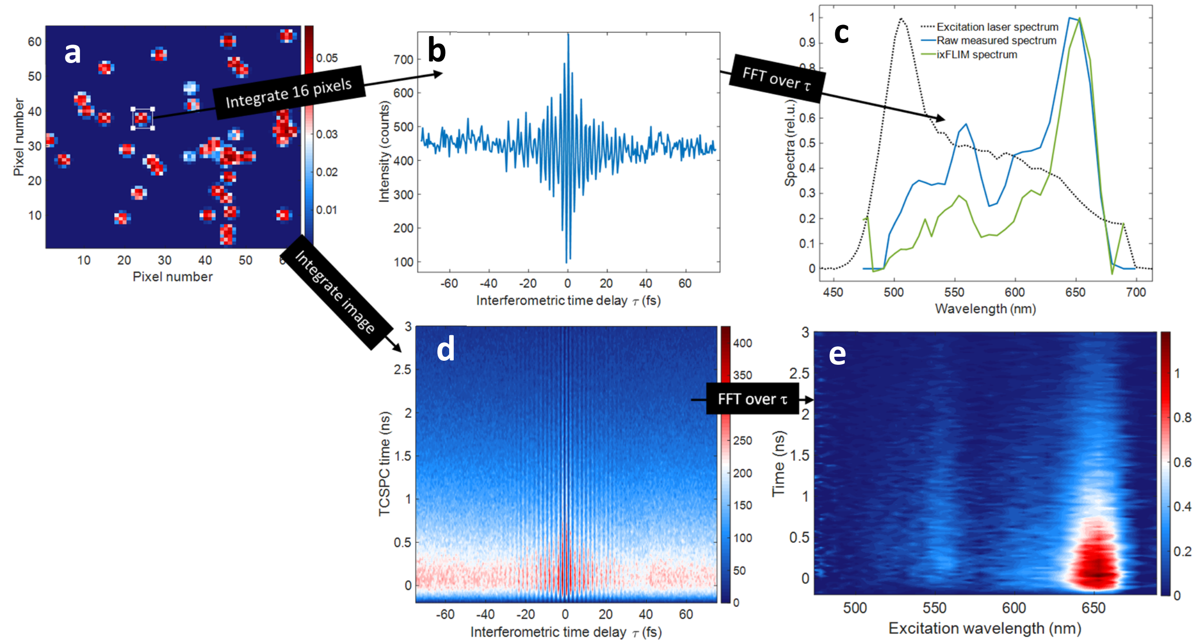

Figure S4 Demonstration of the ixFLIM signal processing. a) image of the bp20 beads binned down to 64x64 pixels. b) TCSPC time-integrated interferogram of a single ball (4x4 pixels), the Fourier transform of which, c), produces the raw spectrum (blue), division of that by the laser spectrum (black) leads to the ixFLIM excitation spectrum (green). d) spatially integrated image produces the time-time correlation map, on x axis is the interferometric time τ, on the vertical axis the TCSPC time delay. e) Fourier transformation along τ produces the ixFLIM transient map.

for which the calibration is correct (for which the step size is indeed 0.75 fs). For the other wavelengths the stage step size is bit different due to dispersion of the wedge crystal. While one can account for the dispersion using the Sellmaier expansion of the refractive index[4], we choose to simply take the maximum point for each wavelength and fit the $\lambda_{FT}(\lambda_{real})$ by a polynomial (in practice linear dependence suffices). Inverting the fit, we get the calibration $\lambda_{real}(\lambda_{FT})$ that we use for the Fourier-transformed ixFLIM dataset. Comparing the spectrum from the Fourier-transformed interferogram (including the $\frac{1}{\lambda^2}$ factor[5]) from Fig. S2b with the spectrum from the spectrometer (Fig. S3b,c), the calibration can be checked.

Next to the calibration, the laser spectrum is used to calculate the ixFLIM data from the raw measured signal as $\mathrm{ixFLIM}(x, y, t, \lambda_\tau) = \frac{\mathrm{ixFLIM_{raw}}(x,y,t,\lambda_\tau)}{\mathrm{spec}(\lambda_\tau)}$.

The procedure of obtaining the ixFLIM data from the measured data is demonstrated in Fig. S4 on the example of the DNA-FRET beads.

S3. Information in ixFLIM: Oxonol VI binding to albumin

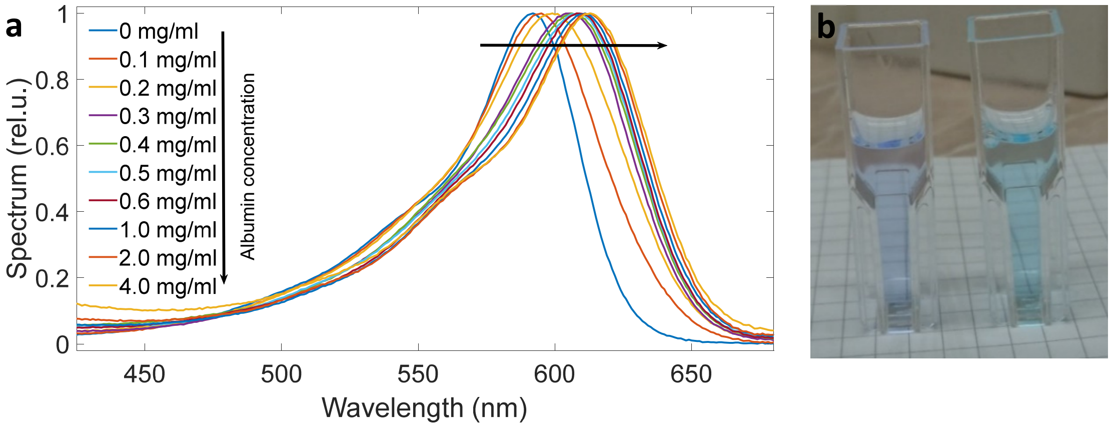

Figure S5 Shift of the Oxonol VI absorption spectrum with bovine serum albumin (BSA) concentration. a) Titration study. Clearly, above 2 mg/ml the solution is saturated, and all Oxo VI is bound to BSA. b) The solutions with free (left, purple) and bound (right turquoise) Oxo VI.

In Fig. S5, the absorption spectrum shift with increasing BSA concentration is shown. The absorption spectra of Oxo VI were measured on absorption spectrometer Varian Cary50 UV/VIS spectrometer. The fluorescence spectra (Fig. 2 in the main text) were measured by Fluoromax spectrometer (Horiba).

Fig S6 shows residues after the global analysis (GA) fit of the oxonol ixFLIM data.

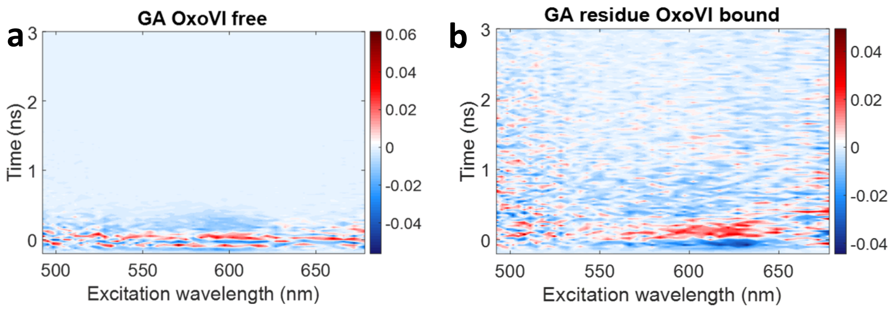

Figure S6 Residues of the global analysis fit of the free (a) and bound (b) oxonol in microcapillaries shown in Fig. 2 of the main text.

S4. FRET molecular ruler: ixFLIM and FLIM on Cy3-Cy5 at DNA

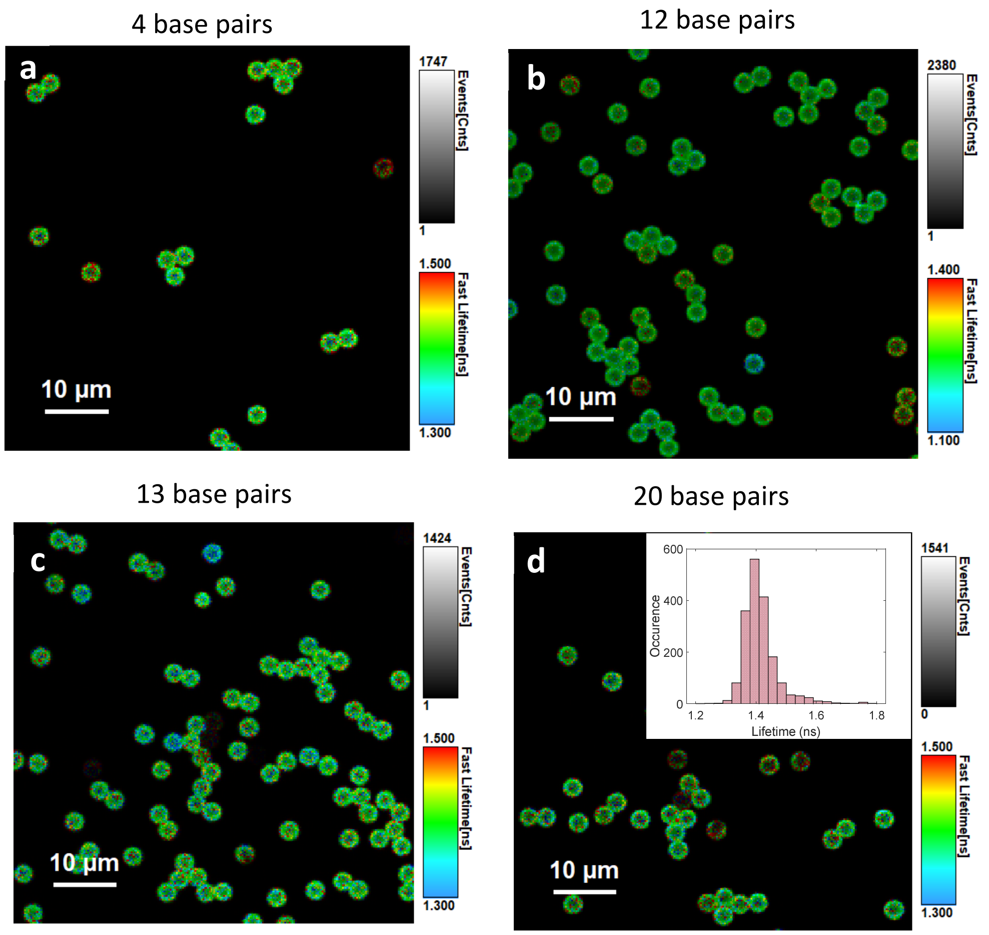

Figure S7 Spectrally-integrated ixFLIM (i.e., FLIM) images of the beads labelled with the DNA constructs. a) to d): Cy3–Cy5 distance of 4 bp, 12 bp, 13 bp and 20 bp. Note the shorter lifetime for the 12 base pair beads. These beads were used for the ixFLIM analysis described here and in the main text. Inset of d) lifetime distribution around 1.4 ns.

Four types of beads with different bound DNA constructs have been measured, with the Cy3 and Cy5 within distance of 4 bp, 12 bp, 13 bp and 20 bp, Fig. S7. While the donor Cy3 position remains fixed, the acceptor position Cy5 varies with the increasing distance. Interestingly, for the 12 base pair position, the Cy5 lifetime is shorter (1.2 ns instead of 1.4 ns), as reflected also by the global analysis (fits below). This can be explained by the variability of the Cy5 quantum yield (and lifetime) dependent on the basis-dependent immediate environment within the DNA[6,7]. The emission lifetimes are narrowly distributed around the acceptor lifetime, 1.4 ns for the bp20 (inset).

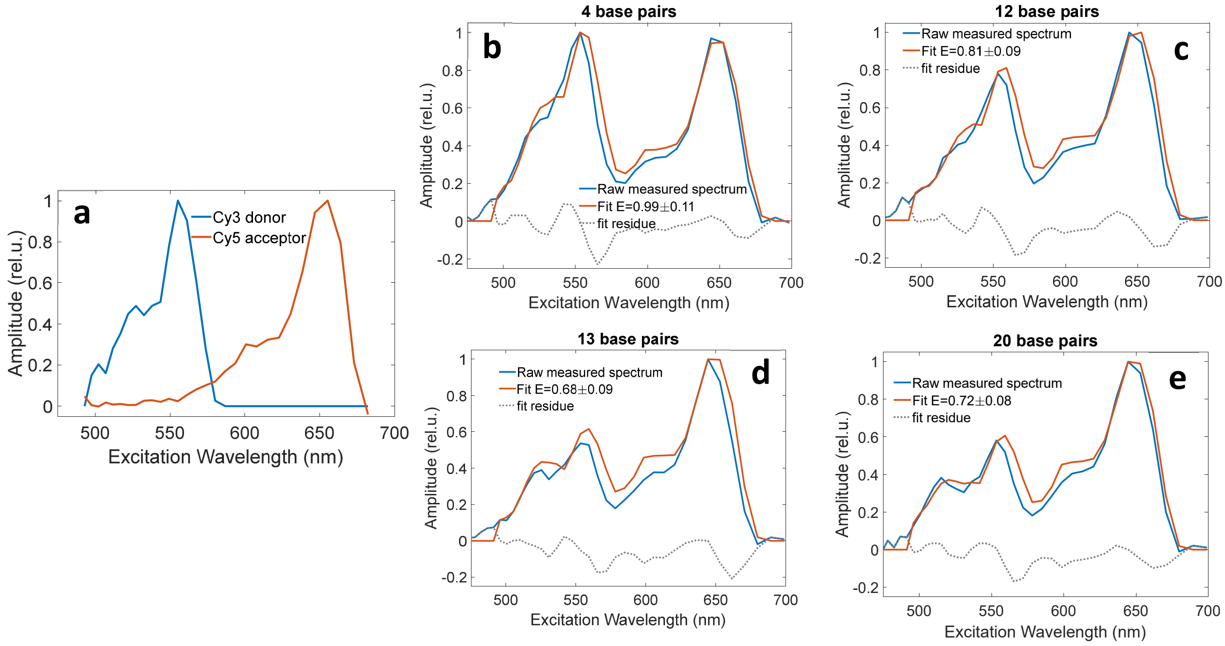

Figure S8 Decomposition of the excitation spectra into those of the donor and acceptor. a) separately measured excitation spectra of the Cy3 donor (blue) and Cy5 acceptor (orange). b) to e), the fits of the raw (not divided by the laser spectrum) $ixFIM(\omega_\tau)$ spectra (blue) by the (laser-spectrum multiplied) weighted sum of the donor and acceptor excitation spectra (orange) according to Eq. 6 in the main text, for bp04, bp12, bp13 and bp20 samples. Fit residues are shown in black dotted lines.

To determine the spectra of the donor and acceptor, we measured beads labelled by only the donor and only the acceptor (Fig. S8a). We used these spectra to fit the excitation spectra of the bp04 to bp20 samples. The ratio of the Cy3/Cy5 extinction coefficients was set to 0.4, in agreement with that obtained by Lee et al. for their 1:1 internally labelled Cy3-Cy5 pairs[8] and to fit the 4 base-pair (13.6 Å) distant donor-acceptor pairs with favorable orientation factor, for which the efficiency is bound to be

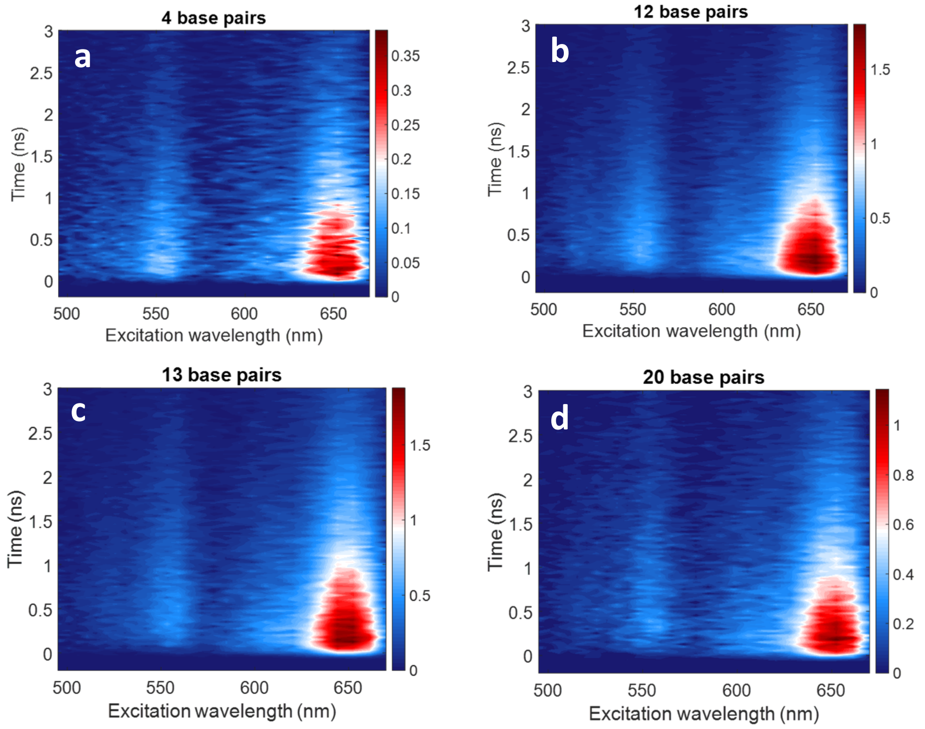

Figure S9 Transient $ixFLIM(t, \lambda_\tau)$ maps for the four Cy3–Cy5 labelled DNA constructs imaged in Fig. S5, a) to d) labels bp04, bp12, bp13 and bp20 samples.

close to unity. This is not only expected by Förster theory with the Förster radius of 71 Å, but also agrees with Ref. 9. Förster theory need not apply to obtain fast transport with efficiency close to unity. The spectra were used for the fitting of the FRET efficiency as described in the Methods section of the main text (Eq. (10) with $c_A = c_D$). To avoid the increase of noise close to the edges of the excitation spectrum by the spectral division, we did not divide the acquired excitation spectra by the laser spectrum, but multiplied the Cy3 and Cy5 absorption spectra instead. The resulting fits for 4 bp, 12 bp, 13 bp and 20 bp distant donor-acceptor pairs, integrated over the beads in Fig. S7, are shown in the panels of Fig. S7b-e. The extracted efficiencies are given in Table 1 and Fig. 4a in the main text. For all the DNA constructs, the whole spatially integrated maps $\mathrm{ixFLIM}(t, \lambda_\tau)$ are shown in Fig. S9. The transient ixFLIM signals were fitted by global analysis using two time components, see Fig. S10. These components correspond to the donor excitation signal rise due to FRET (black) and acceptor emission decay (red). Note the shorter acceptor lifetime for the 12 bp distant dyes, due to the different position of the Cy5 acceptor within the DNA helix. The global analysis was performed in two steps. First, the acceptor-only region (600 nm to 680 nm) was fitted with a single component (see Fig. S12). Then the obtained time constant was fixed and the whole map was fitted with an additional component.

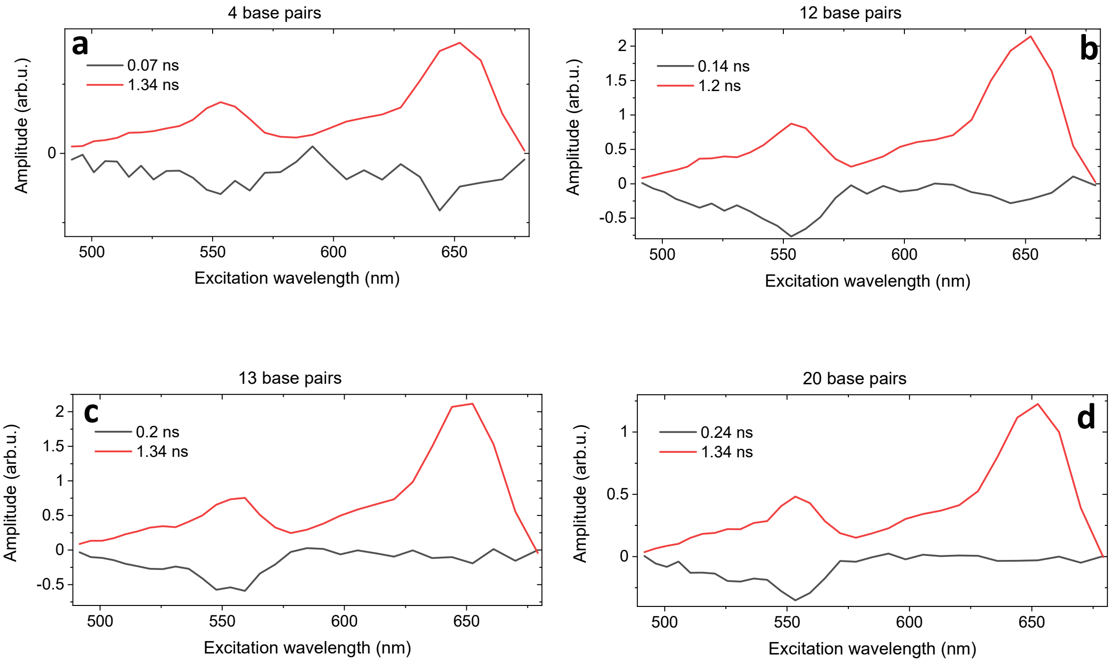

Figure S10 Decay-associated spectra of the two time components from the global analysis of the transient maps shown in Fig. S6. All the recovered rates can be considered with 10% uncertainty, we tested that 10% change in the component time constant leads to only 0.1% change in the fit χ^2 value. a) to d) labels bp04, bp12, bp13 and bp20 samples

The quality of the fits can be judged by the unstructured residues shown in Fig. S11.

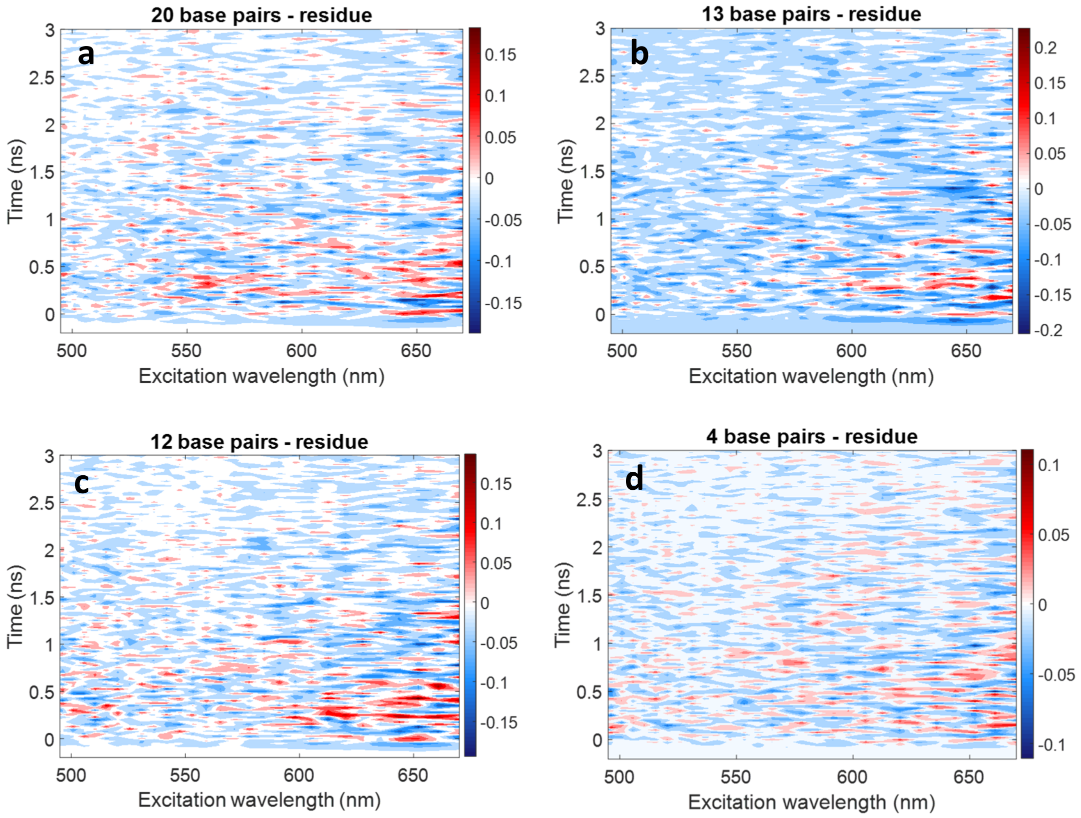

Figure S11 Global-analysis residues from the maps in Fig. S7 fitted with components from Fig. S8. a) to d) labels bp20, bp13, bp12 and bp04 samples.

The lifetime of cyanine dyes is known to change when bound to DNA, dependent on the specific attachment site[6,7]. While the Cy5 acceptor position changes across the constructs, the Cy3 donor attachment remains the same, and thus its lifetime should remain the same as well. We measured the spectrum and lifetime of the Cy3 donor independently, on beads labeled by the single-stranded DNA with the Cy3 donor only (Fig. S12). In the global analysis, we identify two components with the Cy3 spectrum, with time constants of 0.4 ns and 1.27 ns. From the relative areas of these components, we get the average lifetime of $\langle\tau\rangle = 0.75 \pm 0.08\ \mathrm{ns}$. This value is used for k_D^{-1} in the calculation of the transfer time and efficiency from the rising components in Fig. S10. The measured rise time is $k_D + k_T$, and the efficiency is $E = 1 - \frac{k_D}{k_D + k_T}$ as given in Eq. (4) of the main text.

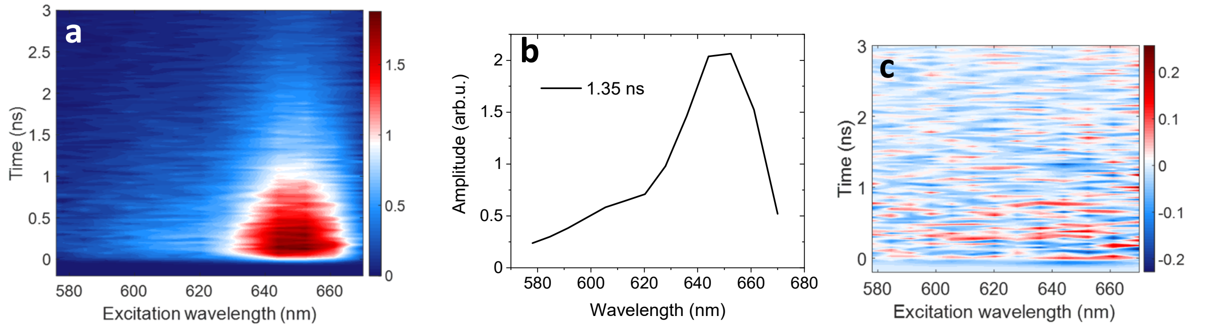

Figure S12 ixFLIM in the acceptor region only (from the b13 data). Since the uphill FRET is insignificant due to the large energy gap, this is equivalent to the free acceptor. a) Transient map, b) single time component used for the global fit, c) global fit residue.

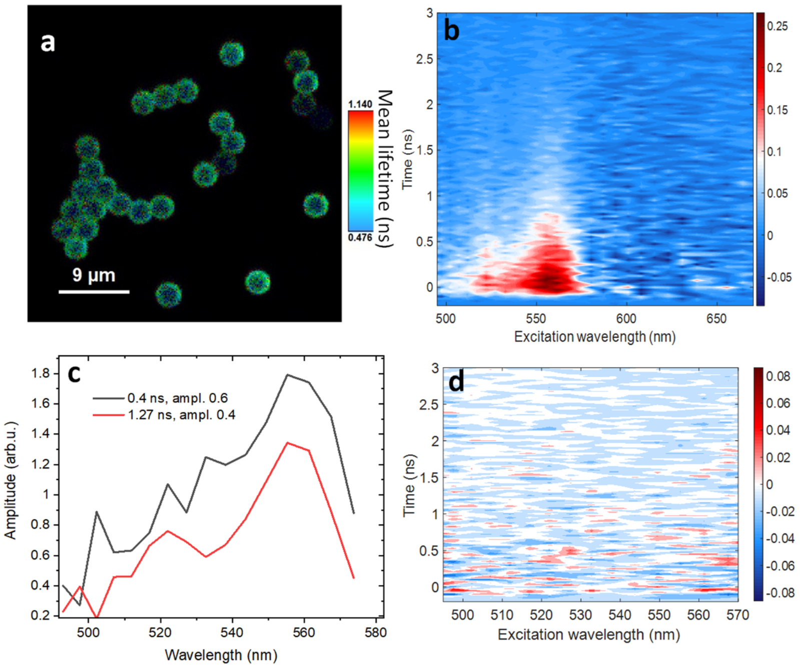

Figure S13 Beads colored by Cy3 only. a) the fluorescence microscope image, b) ixFLIM transient map (detection 600 nm long-pass filter), c) two components obtained from the global analysis fit, d) fit residue.

The spectral fit analysis using Eq. (6) in the main text assumes no direct donor emission is detectable with the long-ass filter. To check this assumption, we measured ixFLIM on the bp20 beads, and then we bleached the Cy5 acceptor using strong 560 nm laser. As the results in Fig. S14 show, the fluorescence intensity drops about 30-fold, the acceptor spectrum disappears, so does the spectrum of the transferring donor, and remains a very weak donor spectrum with amplitude of only couple of percent of the original acceptor signal. The direct donor contribution is thus less than 5% and can be neglected in the analysis. We checked this by taking the donor into account using Eq. (15) in the Methods, getting the same result. This bleaching experiment also validates the same procedure that we used in the standard FLIM measurements, excluding a presence of potential photo-converted Cy5 species.

As another control, we measured unlabeled beads (Fig. S15). These have no red emission and thus are invisible in ixFLIM, and produce a very weak signal in the green upon excitation around 530 nm. Upon excitation at the traditional 488 nm, the autofluorescence is stronger and strongly complicates the FLIM analysis. We thus used a 520-540 nm emission, selected from the broadband PCF white light.

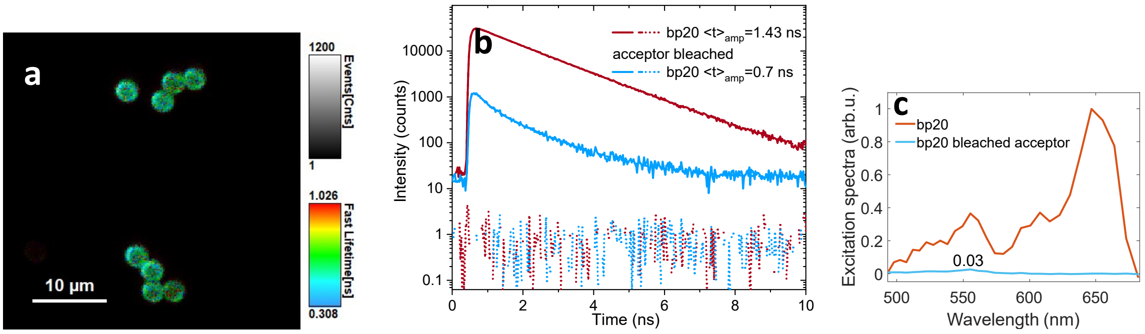

Figure S14 Control ixFLIM measurement on bp20 beads (image in a)), before and after acceptor bleaching. b) While the unbleached sample (red) decays with the 1.4 ns acceptor lifetime, the bleached sample decays with the shorter lifetime of 0.7 ns of the donor (blue). c) The excitation spectra show absorption of both acceptor and donor, after acceptor bleaching both signals disappear and only the donor cross-talk remains, with amplitude of 3%, which is in our analysis negligible.

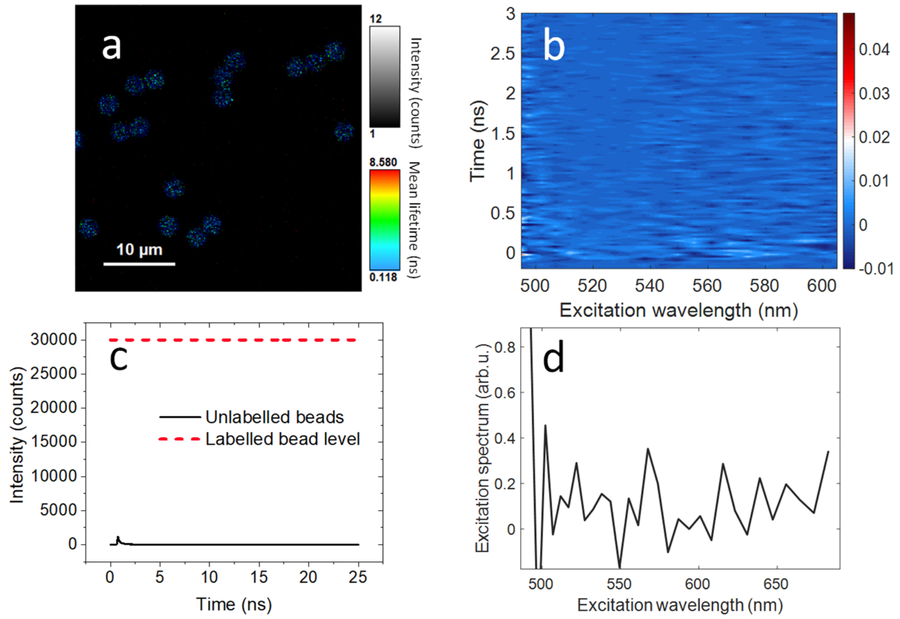

Figure S15 Control ixFLIM and FLIM measurement on unlabeled beads. The beds are near-invisible in red detection, and display weak fluorescence in green (a)). b) ixFLIM data are thus background-free with noisy baseline around zero (c)), while d) FLIM excited at 525 nm and detected in 560 – 600 nm has a weak decaying background that we scaled to the tail and subtracted from the FLIM TCSPC curves before analysis.

In the standard donor FLIM measurements, the decrease of the donor lifetime reports on the excitation energy transfer. In our case, the donor fluorescence decay is not a single exponential, but can be described by at least three exponentials[10]

$$I(t) = I_0 \sum_{i=1}^{3} a_i \, e^{-\frac{t}{\tau_i^D}}.$$

Here, the amplitudes a_i sum to one, and τ_i^D are their associated lifetimes.

The individual components can have a physical meaning, e.g., different conformational states of the Cy3 dye, but the description can be also understood as a mathematical decomposition of the decay. In the presence of the acceptor, the lifetimes of the individual components get shortened by FRET to τ_i^{D-A}

$$I(t) = I_0 \sum_{i=1}^{3} a_i\, e^{-\frac{t}{\tau_i^{D-A}}}.$$

Each of the components has thus a transfer efficiency $E_i = 1 - \frac{\tau_i^{D-A}}{\tau_i^{D}}$. Importantly, the relative fractions of the components remain the same. The total efficiency is the fraction of the transferred donor population,

$$E_{FLIM} = \sum_{i=1}^{3} a_i\, E_i.$$

In the FLIM measurement, we first measured the bead samples with the FRET present. Then we bleached the acceptor with strong 560 nm laser (Obis LS, Coherent) and detected the acceptor emission, until the signal decreased to the noise floor. For the bleaching effects see also Fig. S13, where ixFLIM helps to prove the complete acceptor bleach. We measured FLIM again on the bleached samples, observing increase in intensity and prolongation of the donor lifetime. We performed a joint fit of the bleached and non-bleacheed TCSPC decays with the same relative amplitudes a_i, shown in Fig. S16. We found that three components were needed to fit the non-bleached decays, calculated the individual FRET efficiencies from them, and then the total efficiency. The fit results are summarized in Table S1.

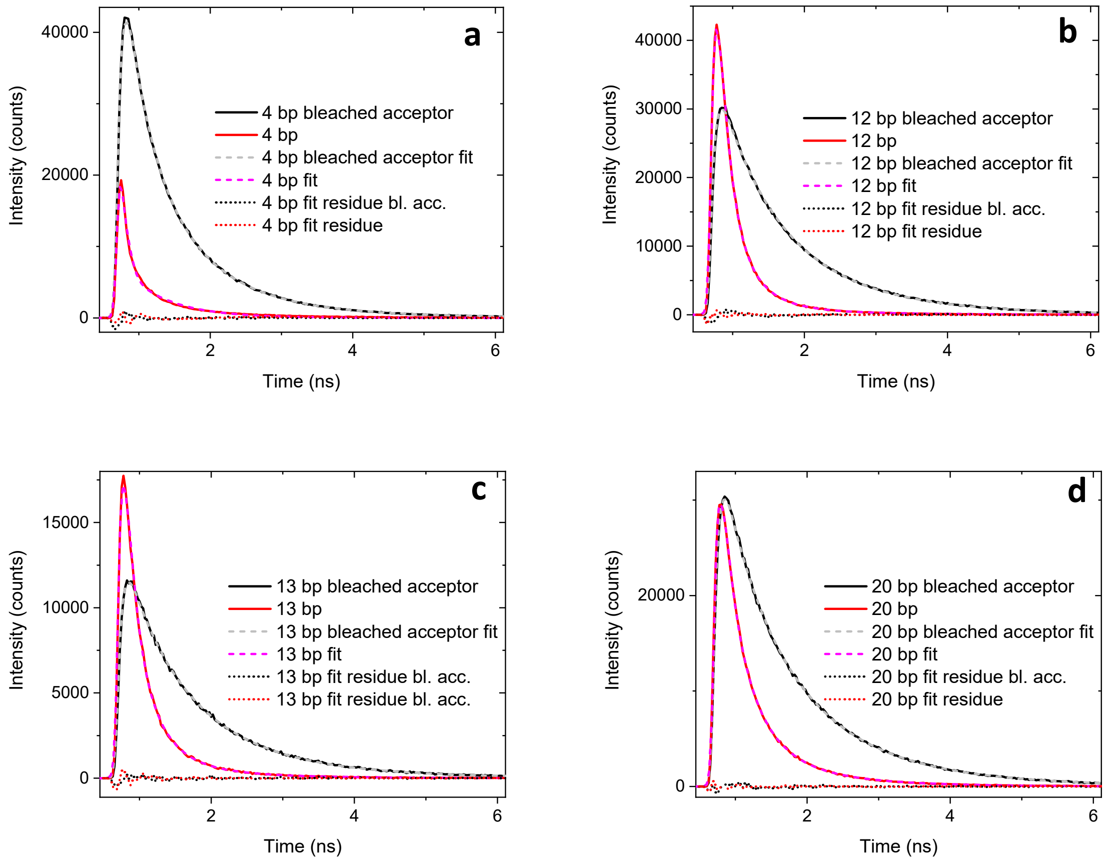

Figure S16 FLIM measurements on the beads with increasing base-pair distance, a) to d) label bp04, bp12, bp13 and bp20 samples. Shown is always FLIM decay curve (red), its fit (pink) and fit residue (red dotted), and FLIM decay with bleached acceptor (black), its fit (grey) and the fit residue (black dots). The fits were done globally using three exponentials with fixed relative amplitudes, re-convoluted with the IRF decomposed into two Gaussians (amplitude 0.66 FWHM 45 ps, amplitude 0.34 FWHM 85 ps, 67 ps apart).

Table S1 Fit parameters of the FLIM measurements

	a1	a2	a3	t1 (ns)	t2 (ns)	t3 (ns)	t1bl (ns)	t2bl (ns)	t3bl (ns)	E1	E2	E3	E
**bp20**	0.29	0.53	0.18	0.13	0.37	1.08	1.69	0.92	0.53	0.25	0.48	0.39	**0.66**
**bp13**	0.53	0.43	0.04	0.13	0.36	1.14	0.87	1.53	0.45	0.89	0.45	0.49	**0.69**
**bp12**	0.71	0.28	0.01	0.15	0.37	1.28	1.28	0.66	0.66	0.81	0.75	0.15	**0.79**
**bp04**	0.42	0.48	0.10	0.005	0.06	0.625	3.00	1.26	0.62	0.99	0.93	0.61	**0.92**

As yet another control, we measured two adjacent beads, one labelled with single-stranded DNA with the donor Cy3 only, and the other with single-stranded DNA with the acceptor Cy5 only, see Fig. S17. In the donor FLIM (530 nm excitation), only the donor-labelled bead is visible, exhibiting the donor lifetime that agrees with that obtained from the global analysis of the separate ixFLIM measurement (Fig. S13). In ixFLIM, majority of the signal comes from the acceptor, with a very weak donor contribution. Imaging by the mean excitation wavelength clearly distinguishes the donor and acceptor beads. ixFLIM map show basically only the acceptor spectrum, with a very weak donor contribution in a faster time component. Crucially, the FRET-associated fast rise component is completely absent, in agreement with the absence of FRET due to the large separation between the dyes on different beads.

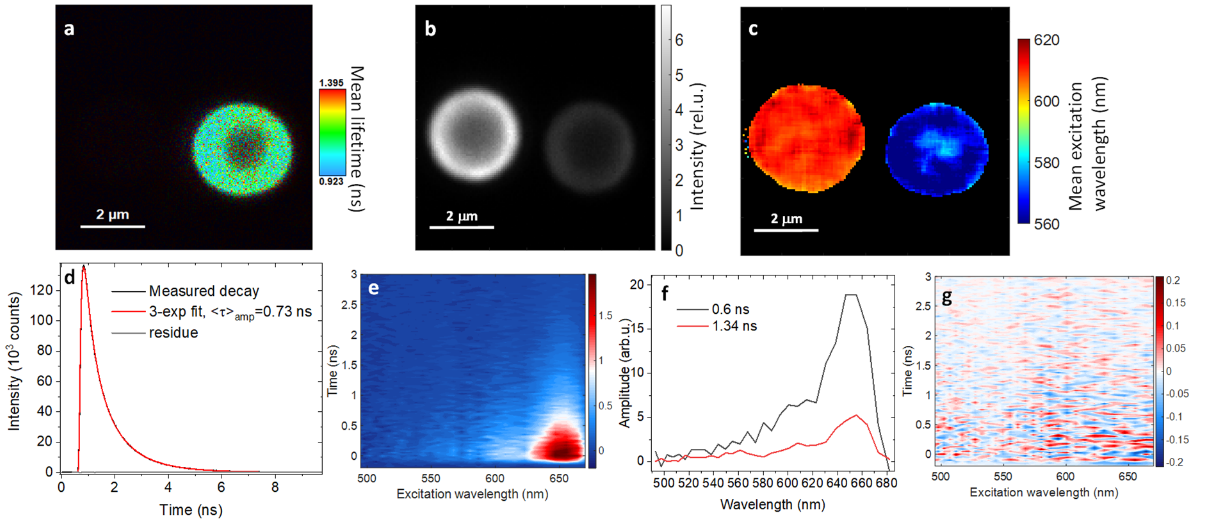

Figure S17 Measurement with two beads 0.5 micrometer apart, one labelled by Cy3 donor and one by Cy5 acceptor. a) FLIM image shows the donor bead only. b) ixFLIM measurement, intensity image showing strong acceptor bead and weak donor bead. c) time-integrated ixFLIM (ixFIM) image clearly distinguishes the donor and acceptor based on their excitation spectrum. d) FLIM decay curve which agrees with the donor lifetime from global analysis in Fig. S13. e) ixFLIM transient map, f) two time components and g) fit residue. The ixFLIM shows almost exclusively the acceptor, with very weak donor spectrum present, but with no negative (i.e., rising) time component. There is thus no FRET taking place, as expected.

In the theoretical FRET model, we take the Cy3 donor and Cy5 acceptor dyes as represented by their transition dipole moments, attached to the DNA double helix on oppposite strands. We take the parameters of the B-type DNA: 10.5 pairs per helix turn, 0.34 helical rise per base pair, 10 Å radius[11]. We consider the dyes to be attached as in Fig. 3a of the main text[8], with a helical rise angle α, azimuthal angle ϕ, and a possible additional distance of the molecule from the helix radius Δ. For the parameters of the DNA-bound Cy3 and Cy5 dyes, we follow Cunningham[9]. Averaging their results for the dye

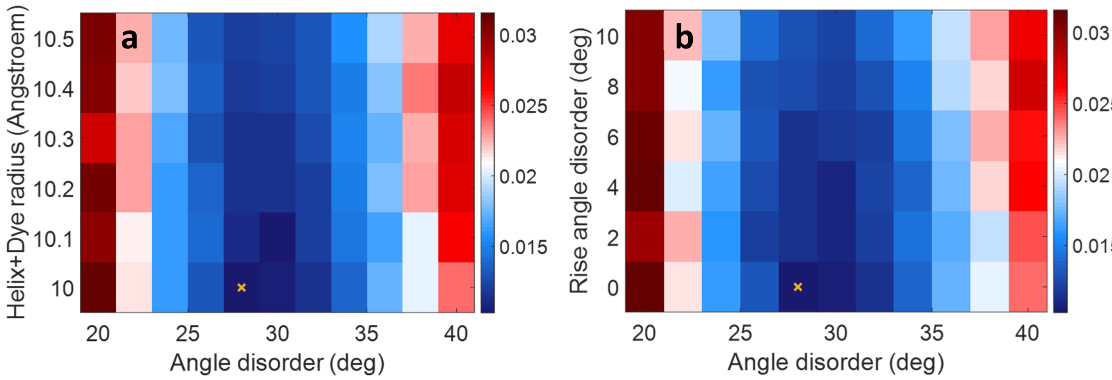

Figure S18 Optimization of the parameters of the Cy3–Cy5 FRET calculation. Mean square deviation as a function of the fit values, cuts for a) fixed $\delta\alpha = 2°$ and b) for fixed $\Delta = 0.1$ Å . Yellow crosses indicate the optimal lowest-MSD points.

distances for which the Foerster regime applies, we get the Foerster radius $R_0 = 71$ Å (assuming random orientation factor of 2/3). In our model, we use the specific donor/acceptor orientation and calculate the Förster rate using formulas (1) and (2) in the main text. The important variables are the inter-dye distance R and their mutual angle, given by $\alpha_{Cy3,Cy5}$ and $\theta_{Cy3,Cy5}$. Due to geometrical fluctuations and the fixed attachment site, the Cy3 and Cy5 angles α, ϕ are taken to be disordered with a Gaussian distribution of angles around their central values given by the fixed helix geometry. For each realization of the angle disorder (taken to be the same for both of the dyes), we calculate the orientation factor $\kappa^2 = \hat{\mu}_a\hat{\mu}_d - 3(\hat{\mu}_a \cdot \hat{R})(\hat{\mu}_d \cdot \hat{R})$ and the inter-dye distance R, and from these the Förster rate. To scale the rate, we calculate also for randomly-oriented dyes at the Förster radius, for which $k_T(R_0) = k_D$ from the definition of the R_0 as the where the FRET efficiency E drops to half, $k_T = k_D \left(\frac{R}{R_0}\right)^6$. For each realization of the disorder, we use this rate to calculate the transfer efficiency $E = \frac{k_T}{k_T + k_D}$, and we also calculate the time-dependent decay of the rising transfer component $S = \frac{k_T}{k_T + k_D - k_A} e^{-(k_T + k_D)t}$. The quantities E and S are then averaged over the normal-distributed angle disorder to produce the average efficiency $\langle E \rangle$ and average signal rise component $\langle 1 - \frac{k_T}{k_T + k_D - k_A} e^{-(k_T + k_D)t} \rangle$. The signal rise is, same as in the experiment, fitted with an exponential to extract the k_T and from this the efficiency is calculated. To compare with the experimental results, the values extracted in these two ways are compared to the experimental values measured form the spectral fit and from the global analysis, calculating the total mean-square deviation MSD between the efficiencies. We globally minimize this $\mathrm{MSD}(\delta\alpha, \delta\phi, \Delta)$ as a function of the disorder in the helical rise angle $\delta\alpha$, disorder of the azimuthal angle $\delta\phi$, and a possible additional distance of the dye transition dipole from the helix radius Δ. The optimal values are $\delta\alpha = 0$, $\delta\phi = 27°$, and $\Delta = 0$ Å. The MSD cuts are shown in Fig. S17 for $\delta\alpha = 0$ (Fig. S18a) and for $\Delta = 0$ Å (Fig. S18b) with the lowest values indicated. These values are used for the theoretical calculation shown in Fig. 4a of the main text, for which the efficiency dependence obtained from the calculated rise and from the rates are averaged. The calculated efficiency dependencies are compared to the experiment in Fig. S19.

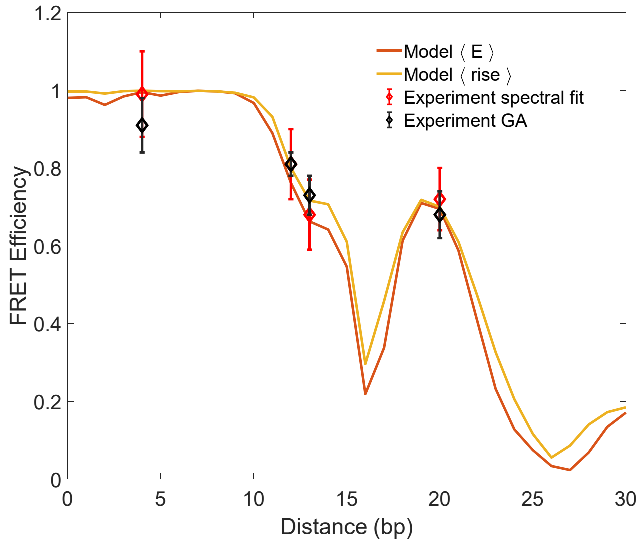

Figure S19 Comparison of the FRET efficiency extracted from spectral fit (red) and from global analysis (black), together with apparent efficiency calculated by averaging the efficiency from rates (orange) and the signal rise (yellow).

Based on the central limit theorem, the distribution of the donor and acceptor angles is typically considered to be normally distributed, as we do in our model. Interestingly, since there are only a few parameters that the FRET rate depends on, the Gaussian angle distribution leads to a highly non-Gaussian distribution of the transfer rates (see Fig. S20). For such distributions, it matters precisely which quantity dependent on the rate is averaged. In the ixFLIM experiment, the total signal with the donor excitation spectrum is described (as derived in the methods section):

$$ixFLIM(\lambda,t) = k_A\phi_A c_D \epsilon_D(\lambda)\sum_{i=1}^{N_{ens}}\left\{\frac{k_T^i}{k_T^i + k_D - k_A}\left(e^{-k_A t} - e^{-(k_T^i+k_D)t}\right) + \frac{\phi_D k_D}{\phi_A k_A}e^{-(k_T^i+k_D)t}\right\}.$$

Here, we included the sum over all transfer rates k_T^i present in the ensemble. We have access to two measured quantities. First, we fit the time-integrated signal in the spectral domain, getting

$$ixFLIM_{Efit} = \phi_A c_D \epsilon_D(\lambda)\left\{\left[\langle E\rangle + \frac{\phi_D\eta_D}{\phi_A\eta_A}(1-\langle E\rangle)\right]\right\},$$

i.e., the efficiency is averaged. The spectral-crosstalk term (second in the square bracket) is very small here (see Fig. S14) and can thus be safely neglected:

$$ixFLIM_{Efit} = \phi_A c_D \epsilon_D(\lambda)\langle E\rangle.$$

Second, we fit (in the global analysis) the time kinetics and focus on the rising component:

$$ixFLIM_{tfit} = -k_A\phi_A c_D \epsilon_D(\lambda)\left\langle\left[\frac{k_T^i}{k_T^i + k_D - k_A} - \frac{\phi_D k_D}{\phi_A k_A}\right]e^{-(k_T^i+k_D)t}\right\rangle.$$

Again, without the spectral cross talk:

$$ixFLIM_{tfit} = -k_A\phi_A c_D \epsilon_D(\lambda)\left\langle\frac{k_T^i}{k_T^i + k_D - k_A}e^{-(k_T^i+k_D)t}\right\rangle.$$

What gets averaged are thus the individual exponential rise components, weighted by a factor dependent on the transfer rate. In effect, fast rises get weight close to one, while the slow ones contribute much less to the total signal. This leads to a small difference between the rates obtained from the rise and from the spectrally integrated fit, as shown in Fig. S19.

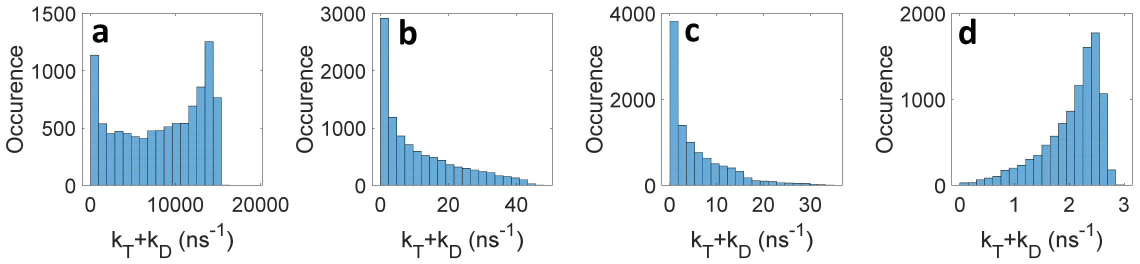

Figure S20 Distribution of FRET rates in the theoretical calculation. Due to the disordered geometry, the distributions are highly non-Gaussian. a) bp04, b) bp12, c) bp13 and d) bp20 sample.

S5. Protein interaction: Nucleophosmin in nuclei and nucleoli of live HEK-293T cells

The NPM interaction was measured for several HEK cells, with very similar results. Since the focus of the paper is presentation of the proof-of-principle ixFLIM measurements and the comparison to FLIM, we show all results on a single representative HEK cell nucleus, shown in Fig. 5 and Fig. 6 of the main text. We analyzed the ixFLIM decays from the whole nucleus, and then separately from the nucleolus and the surrounding nucleoplasm. The transient maps are shown in Fig. S21, together with the residues from the GA fit. On the same nucleus, we measured standard FLIM with 488 nm excitation (PicoQuant). The FLIM image with the two regions defined, together with the corresponding TCSPC decays before and after acceptor bleaching, is shown in Fig. S22.

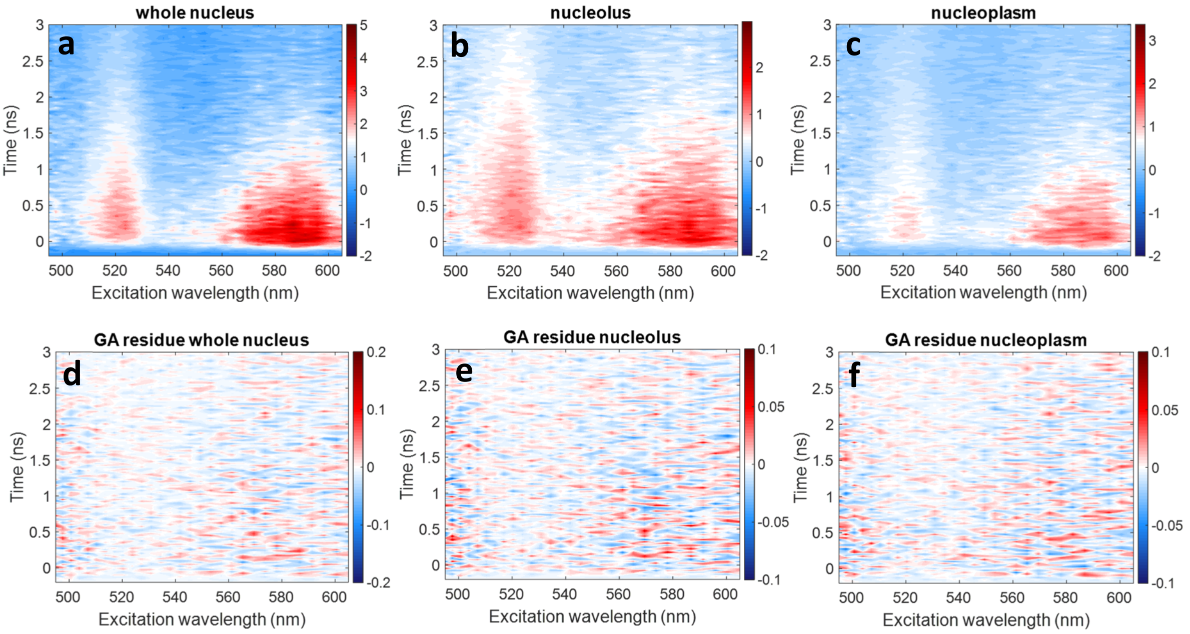

Figure S21 ixFLIM of the interacting NPM in the HEK cell nucleus. a) transient maps of the whole nucleus, b) of the nucleolus only and c) of the surrounding nucleoplasm. d) global analysis residue from the whole nucleus, e) from the nucleolus only, f) from the nucleoplasm. The spectra of the extracted time components can be found in Fig. 5g (whole nucleus) and Fig. 6d (nucleolus and nucleoplasm) in the main text.

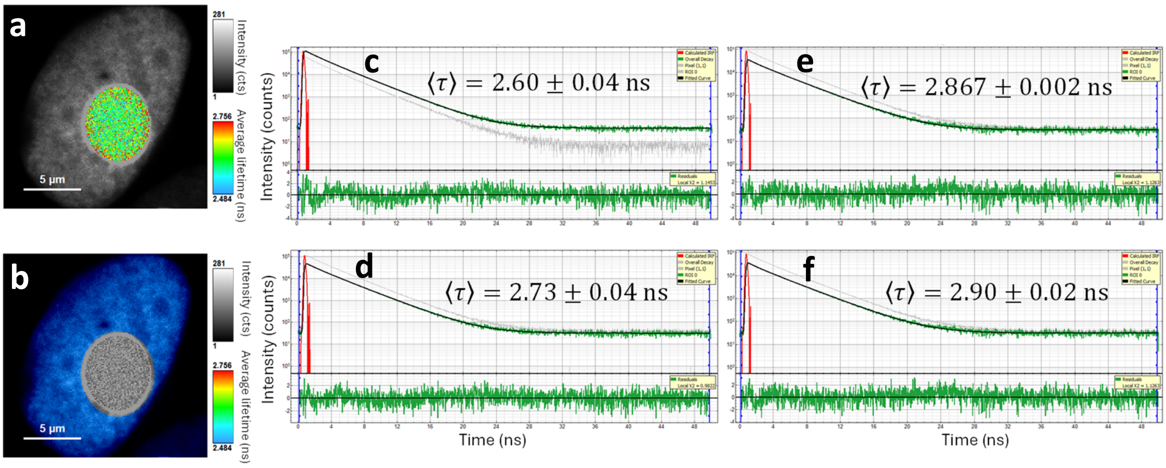

Figure S22 FLIM of the interacting NPM in the HEK cell nucleus. a), b) FLIM images with highlighted regions of interest indicating the nucleolus (a)) and the surrounding nucleoplasm (b)). Decay fits (exported from program Symphotime by PicoQuant) with calculated average lifetimes. Green: TCSPC data, black: multi-exponential fits, red: instrument response function. c) un-bleached nucleolus, d) un-bleached nucleoplasm, e) bleached nucleolus, f) bleached nucleoplasm. Time runs from 0 to 50 ns, vertical lines in 4 ns steps. Bottom: fit residues.

SI References

1. Brida, D., Manzoni, C. & Cerullo, G. Phase-locked pulses for two-dimensional spectroscopy by a birefringent delay line. *Opt. Lett.* **37**, 3027 (2012).
2. Réhault, J., Maiuri, M., Oriana, A. & Cerullo, G. Two-dimensional electronic spectroscopy with birefringent wedges. *Rev. Sci. Instrum.* **85**, 123107 (2014).
3. Dudley, J. M., Genty, G. & Coen, S. Supercontinuum generation in photonic crystal fiber. *Rev. Mod. Phys.* **78**, 1135 (2006).
4. Thyrhaug, E. *et al.* Single-molecule excitation–emission spectroscopy. *Proc. Natl. Acad. Sci.* **116**, 4064–4069 (2019).
5. Mooney, J. & Kambhampati, P. Get the Basics Right: Jacobian Conversion of Wavelength and Energy Scales for Quantitative Analysis of Emission Spectra. *J. Phys. Chem. Lett.* **4**, 3316–3318 (2013).
6. Agbavwe, C. & Somoza, M. M. Sequence-Dependent Fluorescence of Cyanine Dyes on Microarrays. *PLOS ONE* **6**, e22177 (2011).
7. Kretschy, N., Sack, M. & Somoza, M. M. Sequence-Dependent Fluorescence of Cy3- and Cy5-Labeled Double-Stranded DNA. *Bioconjug. Chem.* **27**, 840–848 (2016).

8. Lee, W., von Hippel, P. H. & Marcus, A. H. Internally labeled Cy3/Cy5 DNA constructs show greatly enhanced photo-stability in single-molecule FRET experiments. *Nucleic Acids Res.* **42**, 5967–5977 (2014).
9. Cunningham, P. D. *et al.* Resonance Energy Transfer in DNA Duplexes Labeled with Localized Dyes. *J. Phys. Chem. B* **118**, 14555–14565 (2014).
10. Sanborn, M. E., Connolly, B. K., Gurunathan, K. & Levitus, M. Fluorescence Properties and Photophysics of the Sulfoindocyanine Cy3 Linked Covalently to DNA. *J. Phys. Chem. B* **111**, 11064–11074 (2007).
11. DNA. *Wikipedia* https://en.wikipedia.org/wiki/DNA (2023).
